# 

**Published:** 1920-03-20

**Authors:** 


### TUBERCULOSIS NOTES.

### Welsh Insurance Committees Meet.

A meeting of the above bodies at Swansea was welcomed by the Mayor. In his presidential address, Mr. J. W. Jones referred in complimentary terms to the work of the Welsh National Memorial Association, and welcomed the advent of the Ministry of Health. In the (sixth) annual report exception was taken to the lack of facilities for training discharged consumptive soldiers in suitable occupations. Following the report a feeling manifested itself in the course of a discussion that the Welsh Insurance Committees Association, with only one member upon the Consultative Council, was inadequately represented. A resolution in this sense was agreed to. A medical member urged that severe punishment should be meted out to medical men failing to notify cases of tuberculosis.

Mr. D. W. Evans, director of the Welsh National Memorial, gave some information as to the proposed professorship of tuberculosis at the new Welsh medical school. A scheme was under consideration, he said, for the endowment of a chair, the occupant of which would also be medical adviser to the association. The appointment should be filled by "a man of world-wide reputation, who would bring just that dynamic power to bear which is missing at the present moment."

### Children's Hospital for Johannesburg.

At a meeting held at Johannesburg in February of the General Committee in connection with the proposed Children's Hospital, Lady Buxton, it was resolved to carry out the scheme for building the hospital. A permanent Executive Committee was appointed. It is intended fully to equip a children's hospital containing a hundred beds, to cost between £60,000 and £80,000. The Provincial Administration has promised to maintain the hospital when completed free of further cost to the Johannesburg or Transvaal public.

### Passing Events and Topics.

### Sale of a Hut Hospital.

THE huts at Princess Christian's Red Cross Hospital, Englefield Green, which have been empty for the last six months, have been sold to Egham Urban Council for £5,000, a sum less than half the original cost.

### A Sale of Patients' Work.

THE COUNTESS OF ANCASTER will open a three days' sale of patients' work at the Royal Hospital and Home for Incurables, Putney, on June 8, at 2.30 P.M. The proceeds of the sale are not for the general funds of the hospital, but are devoted wholly to the patients' use as pocket-money.

### Hospital as Health Centre.

THE institution known as Newton Hospital, Massachussetts, comprises the hospital, with an average daily bed occupation of 113, a training school for nurses, a hospital-aid association, a district nursing association, and a social service league. The annual report, which is full of interest, claims that "the hospital should be the health centre for the community."

### Footprint Identification for Hospital Patients.

FINGERPRINTS have long since been one of the means used for the purpose of identifying criminals, but there is something decidedly novel in adapting the plan for the identification of babies in hospital. This, it is stated, has been recently done in New York, where at one children's hospital and day nursery footprints are taken of the little inmates for this purpose.

### A Sermon and Soap.

AN earnest appeal from the pulpit was recently made by a minister of one of the Nonconformist churches in Guildford on behalf of an Indian Medical Mission which was urgently in need at the time of soap supplies, and the congregation, being struck either by the novelty of the appeal or from sympathy for the practical though somewhat pathetic need of their fellow-creatures abroad, contributed no less than 150 bars of that most necessary article.

### A Remarkable Fancy-dress Ball.

THAT interesting London Charity, the Italian Hospital, will share with the Italian Red Cross Open-air Sanatorium in Pozzuoli the proceeds of what promises to be a remarkable fancy-dress ball, which is to be held at Covent Garden on April 28. Chief amongst the organisers are Count de la Feld (Director-General of the General Delegation of the Italian Red Cross, and of London), and Lady Cunard, well known in connection with such social functions. A distinguished artist is preparing a great pageant representing the principal cities of Italy familiarly associated with some of the famous schools of painting. Florence, Venice, Naples, Rome, Verona, and Pisa will each be impersonated by a separate group of dancers in the national and historical costumes of Italy. The first five hundred tickets are to be priced at £2 12s. 6d., including supper, and all tickets over that number will be £3 3s. each.

### St. Mary's Hospital Musical and Dramatic Society.

THE first entertainment of this Society was held in the Medical School on Friday evening, March 5, at 8.30 P.M. The String Trio gave a good opening to the proceedings, a Norwegian dance being well rendered by Miss Best, violin; Miss Hubert, 'cello; and Mrs. Saunderson, piano. The programme included songs from Mr. D. Menzies; a comedy in one act, "Elegant Edward"; and a burlesque melodrama, "Betrayed—and by a Woman." The latter elicited great applause, although some of the technical allusions were lost on the non-medical section of the audience. The Hospital Jazz Band—the last but not least item of the programme—played closing selections. The Hon. Secretaries, Miss Fenwick and Mr. Fry, deserve a hearty vote of thanks for their very hard work for this Society, the *début* of which augurs well for its future.

### King Edward's Hospital Fund for London.

ORDINARY DISTRIBUTION, 1920.

WE are asked to state that hospitals in the County of London, or within nine miles of Charing Cross, desiring to participate in the grants made by this Fund for the year 1920 must make application before the 31st inst. to the Hon. Secretaries, 7 Walbrook, E.C. 4. Applications will also be considered from Convalescent Homes which are situated within the above boundaries, or which, being situated outside, take a large proportion of patients from London. Applications will also be considered from sanatoria for consumption which take patients from London, or which are prepared to place beds at the disposal of the Fund for the use of patients from London hospitals.

### Matrons' and Sisters' Appointments.

City of Westminster Union Infirmary, Fulham Road.—Miss Ethel Oake has been appointed home sister. She was trained at Burnley, and holds the certificate of the Central Midwives Board. She has been sister at Southampton Fever Hospital, ward sister at St. Mark's Hospital, ward and theatre sister at Fulham Infirmary, night superintendent at Hampstead General Hospital, sister at Queen Charlotte's Hospital, night sister at St. James's Infirmary, sister at Bethnal Green Military Hospital, and sister at the City of Westminster Infirmary. Miss Beatrice J. Peters has been appointed second assistant matron. She was trained at Erdington Infirmary, Birmingham, where she was later ward and theatre sister, temporary night sister, and maternity sister. She has since been superintendent nurse at Poole Infirmary, of Guernsey Country Hospital, and night and home sister at City of Westminster Infirmary. Miss Peters holds the certificate of the Central Midwives Board.

Crumpsall Infirmary, Manchester.—Miss Maude M. Shirt has been appointed first assistant matron and Miss Eleanor E. Sealey second assistant matron. They were both trained at Crumpsall, and both hold the certificate of the Central Midwives Board. Miss Shirt was subsequently theatre sister, ward sister, night superintendent, and second assistant matron. She holds the certificate of the Manchester School of Massage. Miss Sealey has been ward sister, night superintendent, and third assistant matron at Crumpsall.

General Hospital, Nottingham.—Miss Isabella Liddle has been appointed assistant matron. She was trained at the Royal Victoria Infirmary, Newcastle-on-Tyne, and has been ward sister, night superintendent, housekeeping sister, and acting assistant matron at Swansea General Hospital.

Meath County Infirmary, Navan.—Miss M. Duffy has been appointed matron. She was trained at the Royal City of Dublin Hospital, has done private nursing, and has been matron of the Severalls House Red Cross Hospital, Newmarket.

Huntly Jubilee Cottage Hospital, Aberdeenshire.—Miss Jessie Spittal has been appointed matron. She was trained at the Edinburgh Royal Infirmary, and has been sister and assistant matron at the Royal Infirmary, Dumfries. She has had five years' war service, two years being passed in military service in Macedonia.

Royal Hospital for Sick Children, Aberdeen.—Miss Winifred Wilson has been appointed sister. She was trained at the Royal Albert Edward Infirmary and Dispensary, Wigan, and at the Baldovan Institution for Mentally Defective Children, Dundee.

Batley and District Hospital.—Miss Clara G. Dawson has been appointed sister. She was trained at the Royal Infirmary, Sheffield.

Johnson Hospital, Spalding.—Miss Ethel Foster has been appointed matron. She was trained at the West Norfolk and Lynn Hospital, where she has since been a member of the private staff, night sister, and acting matron. Miss Foster has also been matron of the Isolation Hospital, Barham, Ipswich.

### Forthcoming Events.

Dr. B. Higgins, medical officer of health for Paddington, is lecturing on "Maternity and Child Welfare" at the Lyceum Club (under the auspices of its Public Service Board) on Friday, March 19, at 4.30 p.m.

London Temperance Hospital, Hampstead Road, N.W. 1.—Forty-seventh Annual Public Meeting, Out-patient Hall, Friday, March 26, 1920, at 5 o'clock, chairman, the Most Hon. the Marquis of Lincolnshire, K.G. Speakers: Sir Alfred Pearce Gould, K.C.V.O., F.R.C.S., M.S., Major Richard Rigg, O.B.E., T.D., J.P., J. Porter Parkinson, Esq., M.D., M.R.C.P., Arthur Evans, Esq., M.S., F.R.C.S., the Rt. Hon. Sir T. Vezey Strong, K.C.V.O., K.B.E.

Medical Officers of Schools Association.—General Meeting, 11 Chandos Street, Cavendish Square, W. 1, on Friday, March 26, at 8 p.m. Dr. G. H. Lock will read a paper on the "Care of Minor Ailments in School Children." All interested are welcome.

### The College of Nursing (Limited by Guarantee).

### EAST LANCASHIRE CENTRE.

The "History of Drugs from the Year 500 B.C." was the subject of a most interesting lecture given by Dr. Leech at the Manchester Royal Infirmary on March 5. About 120 members were in attendance. The continuation of the lecture will be given by Dr. Leech on March 25 at 5.30.

A special general meeting was held after the lecture on March 5, at which Miss Pilgrim presided. The question before the meeting was the nomination of a candidate for the forthcoming Council Election in place of Miss Sparshott, who is due to retire this year. Members were asked to propose the names of candidates they would like to put forward for nomination, and it was unanimously voted that Miss Sparshott should be approached with a view to standing for re-election.

### THE LONDON CENTRE'S DEBATE.

Is Institutional Life Detrimental to Individuality?

The members of the London Centre are to be congratulated upon their first debate, which was held at 11 Chandos Street on Thursday of last week. The subject was one of interest to all nurses—that institutional life is detrimental to the development of individuality—and between seventy and eighty nurses were present. Miss Rundle was in the chair, for the first time, as she carefully explained. The proposer of the motion was Miss Bompas, whose paper was vigorous, humorous, and terse. Her indictment of ward sisters and their part in stunting the growth of personality in their probationers was received with amusement and applause, while her trenchant remarks upon the uniform, especially the cap, of the probationer obviously found an echo in the hearts of her audience. The opposer of the motion was Miss Sheldon, who made an excellent point when she maintained that community life gave a wider sphere for the girl who would otherwise have lived a narrow home life, in which to practise honour, love, judgment, and talent. After the seconders of the speakers, Miss Hall and Miss MacManus, had delivered their speeches, some brisk discussion followed. The vote being taken, it was found that the majority of the audience considered that institutional life was *not* detrimental to the development of individuality. Miss Bagley (Head of the Polytechnic School of Speech Training) then proceeded, as had been previously arranged, to criticise all the speakers, the chairman included, and showed herself a merciless though kindly mimic. Her cold douches must have proved beneficial to the criticised, who from their laughter at their own mistakes appeared to enjoy themselves as much as any of the unmolested.

### The Women's Holiday Fund.

The Annual Meeting of the Women's Holiday Fund, 76 Denison House, Vauxhall Bridge Road, S.W., was held on March 10 at 62 Eaton Place, by kind invitation of the Hon. Mrs. Rennell. The Rev. C. S. Woodward, M.A., Vicar of St. Peter's, Cranley Gardens, presided, and the Countess Ferrers and Dr. Marion Vaughan also spoke on behalf of the Society's work. The Chairman said he hoped it would be possible in the future for every working man to arrange holidays for himself and his family, but that time had not yet come. He therefore urged all who enjoyed the peace and refreshment their annual holidays gave them to support generously a Society which was doing much for the physical and spiritual welfare of some of London's most heroic citizens by providing a fortnight's holiday to tired London working women.

The Wellcome Historical Medical Museum, 54a Wigmore Street, Cavendish Square, W., will be closed for cleaning and redecoration from April 1 to 30 inclusive.

